# Correction of Temporal Hollowing Deformity Using Serratus Anterior Muscle Flap

**DOI:** 10.1155/2022/9523629

**Published:** 2022-01-10

**Authors:** Lan Sook Chang, Youn Hwan Kim, Sang Wha Kim

**Affiliations:** ^1^Department of Plastic and Reconstructive Surgery, College of Medicine, Hanyang University, Seoul, Republic of Korea; ^2^Department of Plastic and Reconstructive Surgery, College of Medicine, Seoul National University, Seoul National University Hospital, Seoul, Republic of Korea

## Abstract

Temporal hollowing deformity (THD) is a contour irregularity in the frontotemporal region, which results in facial asymmetry in the frontal view. Here, we present our clinical experience of correction of THD using serratus anterior (SA) muscle and fascia free flaps. Between March 2016 and December 2018, 13 patients presenting with THD were treated with SA free flap. The mean age of the patients was 47.8 years. The patients received craniectomy due to subarachnoid hemorrhage, epidural hematoma, or brain tumor. On average, correction of THD was performed 17 months after cranioplasty. The SA flap size ranged from 5 × 5 cm to 10 × 8 cm. The mean operation time was 107.3 minutes. All of the flaps survived without complications. The mean follow-up duration was 20.3 months. For correction of THD, the SA muscle and fascia flap is among the best candidates to permanently restore aesthetic form and symmetry.

## 1. Introduction

Temporal hollowing deformity (THD) is a contour irregularity in the frontotemporal region, which is a common complication following surgical dissection of the temporal region or use of the coronal approach for neurosurgery [1–4]. THD is characterized by a concave contour within the pterional region, along the temporal fossa and posterior to the lateral orbital rim, which results in facial asymmetry in the frontal view [2, 5, 6].

Although most cases of THD remain unreported, THD is a highly prevalent complication of craniotomy/craniectomy [7]. The reported incidence is surprisingly high, approaching up to 52%; thus, THD is likely to occur in one of every two patients undergoing craniotomy/craniectomy [5]. Furthermore, THD is permanent even after cranioplasty, which aims only to restore bony continuity without consideration of soft tissue loss during the procedure.

Although the surgical outcome may be functionally successful, THD may cause significant deformity, which can be a source of serious concern and distress to patients due to social stigma [3, 5]. With increased concerns regarding quality of life, there is greater demand for correction of THD [7]. Various techniques and materials have been developed, from bone continuity restoration to soft tissue volume augmentation [1–10]. Autologous fat graft, injection of fillers, autologous tissue transfer, and synthetic alloplastic implants have all been used. Among them, vascularized free tissue transfer has the advantages of deformity correction and maintenance of the soft tissue volume, despite a somewhat unfavorable surgical field [1, 10].

The free serratus anterior (SA) flap has been widely used for reconstruction of the scalp, extremities, and head and neck region. The serratus anterior flap can be harvested when there is an adequate amount of muscle and fascia; adjusting the thickness and dimensions of the flap according to the deformity can promote symmetry. Here, we present our clinical experience regarding correction of THD with an SA muscle and fascia free flap. To the best of our knowledge, this is the first case series using vascularized free tissue transfer for correction of THD.

## 2. Patients and Methods

Between March 2016 and December 2018, 13 patients (9 women and 4 men) presenting with THD were treated with SA muscle and fascia free flaps. The mean age of the patients was 47.8 years (range: 17-74 years). The patients received craniectomy due to subarachnoid hemorrhage (SAH) (*n* = 7), SAH and epidural hematoma (EDH) (*n* = 4), and brain tumor (*n* = 1). Excluding the brain tumor, the etiologies of the injuries were traffic accidents (*n* = 7), aneurysmal rupture (*n* = 3), and falls (*n* = 2). All patients underwent cranioplasty with autologous bone (*n* = 7), poly-methyl-methacrylate (PMMA; *n* = 4), or titanium mesh (*n* = 2).

The demographic and medical history data of all patients, including gender, age, etiology, cranioplasty history, flap dimensions, complications, and follow-up data, were obtained by retrospective chart review (Table 1).

### 2.1. Surgical Technique

Preoperatively, temporal contour deformities were marked with the patient in the sitting position. Under general anesthesia, a longitudinal temporal skin incision 7-10 cm in length was made along the temple hairline. Dissection was performed between the bone and skin according to the dimensions of the THD to create a subcutaneous pocket for flap insertion. An additional preauricular incision was made to expose the superficial temporal vessels. Further dissection along the superficial temporal vessels beneath the parotid gland was often required to obtain adequate recipient vessels. A subcutaneous tunnel was made between the two skin incisions for vascular pedicle passage.

To harvest the SA muscle and fascia flap, patients were placed in the supine position with the arm abducted and elevated. The border of the pectoralis major muscle and the anterior border of the latissimus dorsi (LD) muscle were marked, and a line was drawn along the midportion. A parallel incision was made along the midportion between the anterior border of the LD and pectoralis major muscles. Identification of the anterior border of the LD muscle is essential for successful flap harvesting. Once the anterior border of the LD muscle had been located, the LD muscle was pulled toward the operator. The avascular plane between the LD muscle and the SA muscle fascia was easily dissected, and the subscapular arterial system, including the SA branch, the circumflex scapular vessel branches, and thoracodorsal vessels, could be ligated. Following the SA branch, an outline of the SA muscle and fascia flap was drawn, appropriate to cover the size of the defect. The flap was then harvested in the caudocephalic direction and detached from the ribs. The donor site was closed primarily with negative suction drainage.

The SA flap was carefully positioned and inserted into the subcutaneous pocket, avoiding twisting or curling of the pedicle. The inserted flap was fixed to the fascia or periosteum around the margin of the flap. The pedicle was passed through the subcutaneous tunnel, and end-to-end microanastomosis of the recipient vessels was performed. Postoperatively, prostaglandin analogs were administered intravenously for 2 weeks.

## 3. Results

On average, the correction of THD was performed 17 months (range; 12-24 months) after cranioplasty. The SA muscle and fascia flap size ranged from 5 × 5 cm to 10 × 8 cm. The operation time was 107.3 minutes (range; 75-150 minutes). Additional procedures were performed in three patients at 6 months after the surgery; two patients required additional fat grafts for contour irregularity, and the remaining patient underwent suprabrow lift due to brow ptosis. All of the flaps survived without complications. There was almost no muscle atrophy or flap resorption, so the symmetry of the contour persisted throughout the follow-up period. All donor sites healed without complications, and there were no functional problems. All patients were satisfied with the aesthetic results.

The mean follow-up duration was 20.3 months (range; 12-48 months).

### 3.1. Case Reports

#### 3.1.1. Case 1

A 60-year-old male patient presented with contour depression on the right temple. He had a history of SAH due to a traffic accident and underwent craniectomy. THD remained even at 12 months after cranioplasty using autologous bone (Figure 1). An SA muscle and fascia flap was used for correction of THD.

A 7 cm temporal incision was made along the temporal hairline. A subcutaneous pocket was made by dissection between the skin flap and bone in accordance with the deficient area. An additional incision was made at the preauricular region, and superficial temporal vessels were located. A 10 × 8 cm SA flap was harvested according to the size of the deformity (Figure 2). End-to-end microanastomosis to the superficial temporal vessels was performed. The flap was inserted into the prepared subcutaneous pocket (Figure 3). After 24 months of follow-up, the facial contour of the temporal area was improved, and the patient was satisfied with the aesthetic results (Figure 4).

#### 3.1.2. Case 2

A 37-year-old female patient presented with THD (Figure 5). She underwent craniectomy due to SAH and EDH as a result of a traffic accident and also underwent cranioplasty using titanium mesh 15 months previously. However, THD remained, and she wanted to improve the temporal contour. A 6 × 6 cm SA muscle and fascia flap was harvested. The flap was inserted into the deficient area, and the vascular pedicle was anastomosed to the superficial temporal vessels. The flap survived completely. After 15 months of follow-up, the patient was satisfied with the temporal contour (Figure 6).

#### 3.1.3. Case 3

A 17-year-old female patient presented with THD (Figure 7). She underwent craniectomy due to SAH and EDH as a result of a traffic accident. THD remained even at 18 months after cranioplasty using autologous bone. An SA muscle and fascia flap was planned for correction of THD. An 8 × 7 cm SA muscle and fascia flap was harvested. The flap was inserted into the deficient area, and the vascular pedicle was anastomosed to the superficial temporal vessels. The flap survived completely. After 48 months of follow-up, the patient was satisfied with the temporal contour (Figure 8).

## 4. Discussion

Unilateral THD occurs most commonly as a consequence of neurosurgery. Exposure procedures during craniotomy/craniectomy lead to temporalis muscle atrophy due to muscle injury, vascular injury, and nerve denervation [11–13]. Therefore, THD remains unchanged after cranioplasty, which only restores bone continuity [2, 5]. Another challenge when correcting THD is its unpredictable onset (ranging from early, i.e., within a few weeks, to several months after injury) and the progressive nature [14, 15]. THD is often identified clinically 6 months after the surgery [13] and worsens with time due to progression of atrophy [6]. In addition, as THD is due to temporal muscle atrophy rather than bone deformity, it is difficult to perform objective measurement of THD by radiologic study. However, it is obvious in frontal facial photography of the patient (Figures 1, 5, and 7), which is a serious concern and distress to patients.

To correct persistent THD even after cranioplasty, various techniques using autologous and alloplastic materials for soft tissue augmentation of temporal regions have been introduced, including autologous fat graft, injection of fillers, autologous tissue transfer, and implantation of alloplastic materials, such as PMMA.

Alloplastic materials made of various materials, such as PMMA implants, titanium mesh implants, and high-density porous polyethylene implants (Medpor; Stryker, Kalamazoo, MI, USA), are widely used in secondary or revision cranioplasty [3, 16–19]. However, alloplastic materials always pose a risk of infection, hematoma, and foreign body reaction [1]. The infection rate associated with alloplastic materials has been reported to range from 0% to 6.3%, with an overall complication rate of 24.6% for PMMA implants [1, 20]. These materials are used to ensure bony continuity, rather than to address soft tissue deficiencies.

Autologous fat grafts and filler injections are most commonly used for correction of THD [21, 22]; they are technically simple, have minimal or no donor site morbidity, and can be performed under local anesthesia in the outpatient clinic. However, they show unpredictable resorption, as the temporal region has decreased vascularity due to previous surgical procedures; therefore, repeated procedures may be required [6, 23]. Moreover, there have been reports of catastrophic complications, such as fat embolism, stroke, blindness, and even death after fat graft or filler injection [24–28].

The goal of THD correction is to permanently restore aesthetic form and symmetry. Vascularized free tissue transfer provides sufficient soft tissue volume to correct THD permanently, and the robust blood supply ensures volume augmentation effect even in an irradiated or contaminated surgical field. The disadvantages of free tissue transfer include its complexity, long surgical time and hospitalization period, and donor site morbidity. Therefore, there are only two case reports using free tissue transfer to correct THD [1, 10].

The SA free flap was first reported by Takanayagi and Tsukie [29] and has been widely used for reconstruction of the scalp, extremities, and head and neck region [30–34]. Based on thoracodorsal vessels, the SA flap is known to have consistent anatomy, and long vascular pedicle; moreover, it is easy to harvest and has minimal donor site morbidity [34]. Therefore, SA muscle and fascial flaps can be used for correction of THD, and overcome the disadvantages associated with free tissue transfer. First, harvesting of the SA flap is quite simple and straightforward. By pulling the LD muscle, the avascular plane between the LD and SA muscle can be easily dissected. On identification of the subscapular arterial system, the SA branch can be followed easily to the SA muscle, which is attached to the ribs. The SA muscle and fascia flap can be harvested without the need for tedious intramuscular dissection. Second, the flap matches the size and dimensions of the THD. The SA muscle is one of the thinnest and smallest muscles in the body. The SA flap can be harvested if there is an adequate amount of muscle and fascia; moreover, the thickness and dimensions of the flap can be adjusted according to the deformity. Third, microanastomosis is straightforward and has a reliable vascular supply. For the vascular pedicle, the SA branch joins the thoracodorsal artery, and a long, large-diameter vascular pedicle can be obtained with reliable vascular flow. The thoracodorsal artery is familiar to most microsurgeons who have the experience with LD muscle flaps. End-to-end microanastomosis to the superficial temporal artery, which matches the diameter and thickness of the thoracodorsal artery at the preauricular area, is straightforward.

Given the ease of harvesting and microanastomosis, the surgical procedure is relatively short compared to other types of free tissue transfer. In our case, the surgery took a mean of only 107.3 minutes (range; 75-150 minutes), which was considerably faster than procedures involving other muscle or perforator flaps. Although the vascularized free flap procedure is complex and requires considerable surgical skill, given the relatively short operation time and permanent aesthetic improvement, the use of the SA muscle and fascia flap can be a good option.

There are several concerns related to free tissue transfer, where autologous material can be problematic in terms of donor site morbidity. Fortunately, the SA muscle has minimal donor site morbidity compared to other muscle flaps. The donor site can be closed and is hidden at the midaxillary line, which is less exposed. The need for an additional skin incision along the temporal hairline, and an incision in the preauricular area, should be explained to the patient prior to surgery.

Another concern is the availability of a reliable recipient artery. As most of our patients had undergone several neurosurgical procedures, the availability of a superficial temporal artery is not guaranteed. However, a superficial temporal artery is always present in the preauricular area, often beneath the parotid gland. At this location, the superficial artery has a similar diameter and thickness to the thoracodorsal artery, which facilitates microanastomosis.

Muscle atrophy due to resorption can be a concern with respect to long-term maintenance of the soft tissue volume. The SA muscle is harvested together with its fascia, which can lend rigid support and provide a chain-like blood supply, to the muscle. A healthy, well-vascularized muscle and fascia flap can overcome ischemic conditions in the temporal region and maintain the correct volume permanently. The volume and dimension of the harvested SA muscle and fascia flap were maintained throughout the follow-up period in this study. Only two patients required additional fat grafting for better aesthetic results.

This study had several limitations including a retrospective design, relatively small number of patients, and absence of a control group. Further studies on the thickness and soft tissue volume outcomes of THD correction are required.

For correction of THD, the SA muscle and fascia flap can be a good candidate to permanently restore aesthetic form and symmetry.

## Figures and Tables

**Figure 1:**
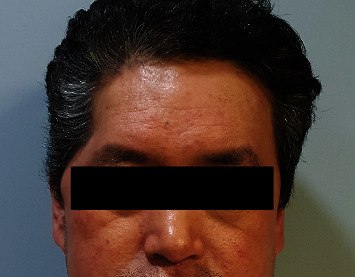
A 60-year-old male patient presented with contour depression on the right temple.

**Figure 2:**
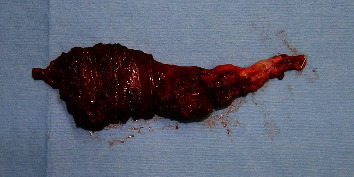
A 10 × 8 cm SA flap was harvested in accordance with the size of the deformity.

**Figure 3:**
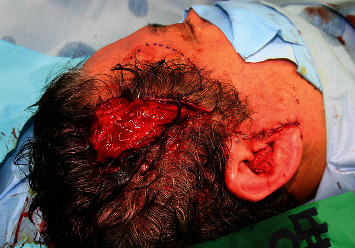
End-to-end microanastomosis was performed to the superficial temporal vessels through preauricular incision. The flap was inserted into the prepared subcutaneous pocket.

**Figure 4:**
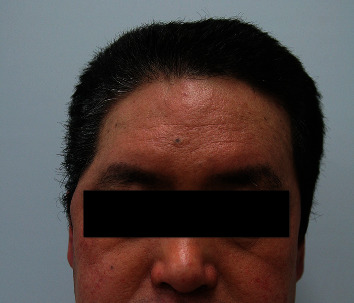
At the 24 month follow-up, the facial contours of the temporal area were maintained.

**Figure 5:**
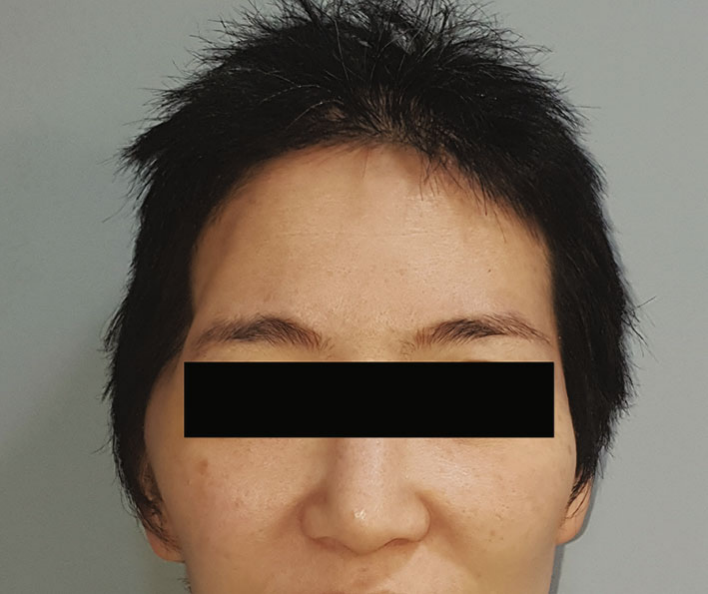
A 37-year-old female patient presented with temporal hollowing deformity.

**Figure 6:**
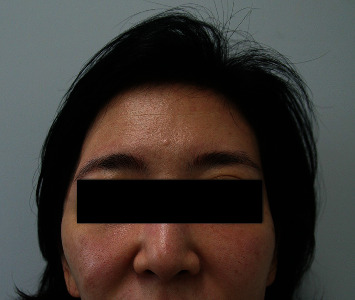
At the 15-month follow-up, the patient was satisfied with the temporal contours.

**Figure 7:**
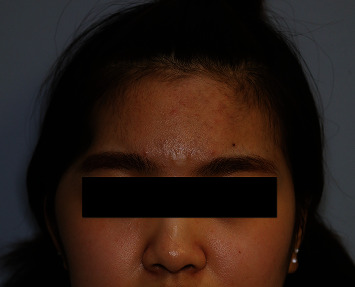
A 17-year-old female patient presented with temporal hollowing deformity on the right temple.

**Figure 8:**
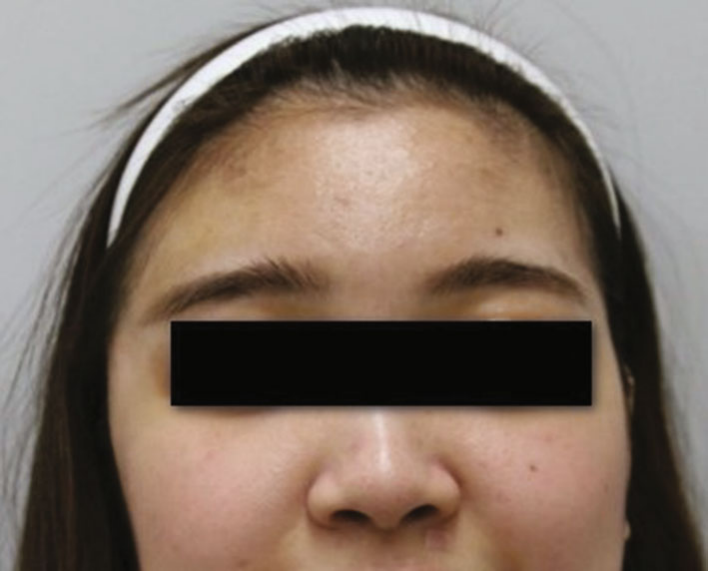
At the 48-month follow-up, the patient was satisfied with the temporal contours.

**Table 1:** Patient information.

No.	Sex	Age	Cranioplasty material	Etiology of craniectomy	Etiology of injury	Time after cranioplasty	Flap size (cm^2^)	Additional procedure	Operative time (min)	Follow-up (months)
1	M	60	Autogenous bone	SAH	TA	12	10 × 8	None	75	24
2	F	74	Autogenous bone	SAH	Fall	18	7 × 5	None	90	15
3	F	65	Autogenous bone	SAH	Fall	24	9 × 6	None	80	18
4	M	57	PMMA	SAH	Aneurysm rupture	24	6 × 6	Suprabrow lift	120	18
5	F	41	Autogenous bone	SAH	TA	15	5 × 5	None	100	18
6	F	17	Autogenous bone	SAH, EDH	TA	18	8 × 7	Fat injection	120	48
7	M	54	PMMA	Brain tumor	None	12	10 × 5	None	110	24
8	F	44	Titanium mesh	SAH	Aneurysm rupture	12	10 × 7	None	120	12
9	F	53	PMMA	SAH	Aneurysm rupture	12	5 × 5	None	90	18
10	F	37	Titanium mesh	SAH, EDH	TA	15	6 × 6	Fat injection	90	12
11	F	51	Autogenous bone	SAH, EDH	TA	15	7 × 6	None	100	12
12	F	26	Autogenous bone	SAH	TA	24	8 × 6	None	150	30
13	M	43	PMMA	SAH, EDH	TA	20	10 × 8	None	150	15

SAH: subarachnoid hemorrhage; EDH: epidural hematoma; TA: traffic accident.

## Data Availability

The data used to support the findings of this study are available from the corresponding author upon request.
